# Liquid chromatography versus supercritical fluid chromatography coupled to mass spectrometry: a comparative study of performance for multiresidue analysis of pesticides

**DOI:** 10.1007/s00216-021-03565-4

**Published:** 2021-07-29

**Authors:** Víctor Cutillas, Carmen Ferrer, Amadeo R. Fernández-Alba

**Affiliations:** European Union Reference Laboratory for Pesticide Residues in Fruit & Vegetables, University of Almeria, Agrifood Campus of International Excellence (ceiA3), Ctra. Sacramento S/N, La Cañada de San Urbano, 04120 Almería, Spain

**Keywords:** Supercritical fluid chromatography, Reverse-phase, Liquid chromatography, Mass spectrometry, Pesticide analysis

## Abstract

Abundant studies have been published evaluating different parameters of reverse-phase liquid chromatography (LC) and supercritical fluid chromatography (SFC), both coupled to electrospray (ESI)/mass spectrometry (MS) for pesticide residue analysis. However, there is a lack of a comprehensive comparative study that facilitates deep knowledge about the benefits of using each technique. In the present study, the same mass spectrometer was used coupled to both liquid and supercritical fluid chromatographies with a multiresidue method of 215 compounds, for the analysis of pesticide residues in food samples. Through the injection of the spiked extracts, separate experiments were conducted. A study of the optimum ion source temperature using the different chromatography modes was performed. The results were evaluated in terms of sensitivity with tomato, leek, onion, and orange as representative fruit and vegetable matrices. The compounds which reported the highest area values in each chromatography were evaluated through their substance groups and polarity values. The impact of matrix effects obtained in tomato matrix was similar for both cases; however, SFC clearly showed better results in analyzing matrices with a higher number of natural co-extracted compounds. This can be explained by the combination of two effects: (i) chromatography separation and (ii) ion source efficiency. The chromatographic elution presented different profiles of matrix components, which had diverse impact on the coelution with the analytes, being more beneficial when SFC was used in the matrices studied. The data showed that the best results obtained in SFC are also related to a higher ionization efficiency even when the ESI emitter tip was not optimized for SFC flow. In the present study a comprehensive evaluation of the benefits and drawbacks of these chromatography modes for routine pesticide residue analysis related to target compounds/commodities is provided.

**Graphical abstract:**

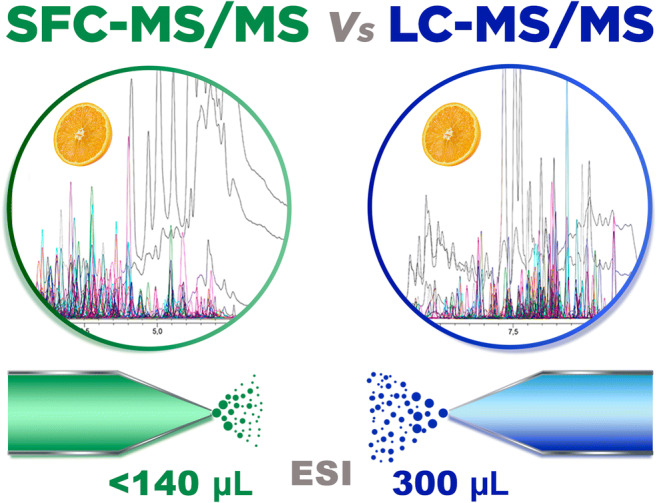

**Supplementary Information:**

The online version contains supplementary material available at 10.1007/s00216-021-03565-4.

## Introduction

Since the introduction of modern mass spectrometer devices, reverse-phase liquid chromatography (LC) has been used as the prevailing technique for the analysis of relatively polar and thermolabile pesticides [1, 2]. The system robustness and the broad scope covered made it one of the preferred approaches to work coupled to mass spectrometry in routine laboratories. On the other hand, supercritical fluid chromatography emerged in 1962 as a technique with much potential; however, the drawbacks and limitations were high due to the lack of devices necessary to perform an efficient analysis. During the consecutive decades, many milestones helped improve SFC performance [3, 4]. Numerous studies highlighted the kinetic performance of SFC like the enhanced linear velocity compared to liquid chromatography. This fact can be easily observed through the van Deemter diagram, where the particular properties of the supercritical fluid provide an improved diffusion coefficient and solvent strength compared to LC [5]. In SFC, the elution profile is directly related to the density of the mobile phase; for this reason, the development of an efficient back pressure regulator (BPR) device that allows keeping the outlet pressure at the desired value is one of the most essential achievements in the SFC evolution [6]. The different couplings of the BPR in the SFC system have been studied since its implementation [4, 7]. Apart from that, the use of a co-solvent for carbon dioxide during the elution ushered a wide range of possibilities [8, 9]. The use of modifiers can favor the solubility of some compounds, improving chromatographic quality. Furthermore, the use of additives in the modifier enhances the interactions with the column stationary phase and the pH stability of the mobile phase [9]. Apart from the high number of possible combinations for the mobile phase in SFC, the interactions with the stationary phases usually present differences compared to LC. West et al. analyzed 109 compounds using 31 distinct stationary phases with the aim of facilitating the column selection in SFC [10]. The numerous advances and achievements in the last decades provided SFC with the necessary quality to be an alternative to the conventional liquid chromatography.

Supercritical fluid chromatography coupled to mass spectrometry has been used for the analysis of compounds in different fields, including environmental and food analysis. The online extraction coupled with SFC-MS/MS provides a fast and reliable determination of natural products like carotenoids and apocarotenoids in food and biological matrices [11]. This type of extraction is also hyphenated to SFC-MS/MS for the analysis of polycyclic aromatic hydrocarbons (PAHs) in soil matrix [12]. SFC coupled to high-resolution mass spectrometry (HRMS) provides a robust analytical method for the simultaneous quantification of persistent and mobile organic substances [13]. Moreover, this type of hyphenation of HRMS with SFC is often used in the metabolomic field where the matrix effect reduction seems to improve the analysis in complex matrices like urine or plasma [14]. However, one of the most important applications of supercritical fluid chromatography is the analysis and quantification of stereoisomers in chiral compounds [15]. In the last decades, this enantiomer separation with SFC has remarkably increased in the pharmaceutical industry [16]. Furthermore, preparative LC is being replaced by preparative SFC due to the advantages of using carbon dioxide as the mobile phase: high flow rate, short equilibration time, no dilution effect, and lower solvent consumption [17].

Regarding pesticides, the interest in using SFC to detect and quantify these residues has increased in recent years [18]. Some evaluations of multiresidue methods have been performed using tandem mass spectrometry [19, 20] and HRMS [21, 22]. As pesticides are often chiral compounds, enantioseparation of pesticides by SFC-MS/MS was previously researched [23–25]. Since the commercialization of ultra-high performance supercritical fluid chromatography, there is an increasing tendency to use this system for food analysis applications.

The advantages of SFC-MS/MS have been profusely described: low matrix effects, short run times, lower solvent consumption, etc. However, there is not a vast number of bibliographic references about direct comparison of SFC with reverse-phase LC in pesticide residue analysis. The main objective of the present paper is to compare the results of the analysis of 215 pesticides using SFC-MS/MS and LC-MS/MS through different experiments. Both types of chromatography were performed with the same mass spectrometry platform. BPR and CO_2_ pump devices were disconnected while using the LC mode. The configuration remained the same, except for the mobile phase composition and column length (SFC 25 cm, LC 15 cm). The same extracts were injected on the same day on both platforms. The sensitivity using both chromatographic tools was compared through different ESI temperatures and different matrices analyzed. Matrix effects were also evaluated. The results of these experiments can help routine laboratories select the technic which fits better with their scope.

## Materials and methods

### Reagents and materials

The standards of pesticides included in the multiresidue method of study were provided by LGC (Teddington, UK) and Dr. Ehrenstorfer (Augsburg, Germany). The analytical standards were stored at −30°C. The standard-mix solution was prepared using individual stock solutions. Individual stock solutions (800–1000 mg/L) were prepared from each standard and stored in the dark at −30°C in amber glass vials.

Carbon dioxide (CO_2_) 5.3 quality was supplied by Abello Linde (Madrid, Spain); LC-MS quality methanol used for mobile phase preparation was obtained from Fluka Analytical (Steinheim, Germany). LC-MS grade water from Fisher Chemical (Fair Lawn, NJ, USA) was used. The additives ammonium formate and formic acid were purchased from Sigma Aldrich (Steinheim, Germany). The salts employed in the QuEChERS extraction (anhydrous magnesium sulphate, sodium chloride, sodium hydrogenocitrate sesquihydrate, and sodium citrate tribasic dihydrate) were supplied by Sigma-Aldrich (Steinheim, Germany) except for PSA that was obtained from Supelco (Bellefonte, PA, USA).

### Sample preparation

The four matrices studied (tomato, onion, orange, and leek) were obtained from a local market in Almería (Spain). These matrices were extracted by Citrate buffered QuEChERS with dispersive solid phase extraction (dSPE) cleanup [26] and analyzed to ensure that they did not have any detectable pesticide residue that could interfere with the study. The final extract resulting from the extraction method contained 1 g of matrix per milliliter. Matrix-matched vials were prepared by evaporating 100 μL of each blank extract under a gentle stream of nitrogen and reconstituted with the same volume of acetonitrile containing the mixture of the analyzed pesticides at the desired concentration.

### Supercritical fluid chromatography

The SFC analysis was performed using a Nexera UC (Shimadzu Corporation, Kyoto, Japan). In addition to the standard devices of liquid chromatography (binary pumps, column oven, and autosampler), this system was equipped with a CO_2_ pump and a back-pressure regulator (BPR) splitless device just before the MS source.

Methanol with 1 mM ammonium formate was used as a co-solvent (modifier) and mixed with the carbon dioxide during the gradient. The make-up solvent used after the column was methanol containing 5mM ammonium formate, 0.1% formic acid, and 5% of water. The make-up solvent was introduced in the system isocratically at 0.080 mL/min. The SFC separation was carried out on a C18 stationary phase column Shimpack UC-X RP (3μm, 250 × 2.1 mm). The oven temperature was set at 40 °C. The BPR pressure and temperature were established at 150 bar and 50 °C, respectively. The total flow used was kept constant at 1.3 mL/min and 2μL was the injection volume. The gradient used during the analysis was as follows: an isocratic flow of 1% of modifier was kept for 2 min; the modifier percentage increased linearly to 4% at min 4 and was increased to 8% at minute 8. After 1 min, the modifier percentage increased to 40%, and this condition was kept for 2 min. The modifier percentage was then reduced from 40 to 1% to recover initial conditions and maintained over 2 min.

### Liquid chromatography

The LC analysis was performed using the same system described above but avoiding the use of the CO_2_ pump and the BPR. The LC separation was carried out on a C18 stationary phase column Shimpack UC-X RP (3μm, 150 × 2.1 mm). The mobile phase flow used was 0.3 mL/min, and the injection volume was the same as in SFC (2μL). The mobile phase A was 98% water and 2% methanol, whereas mobile phase B was 98% methanol and 2% water. Both mobile phases contained 5 mM ammonium formate and 0.1% formic acid. The mobile phase gradient started with 100% of mobile phase A. It was reduced to 75% at minute 1.5 and to 50% at minute 2.5. After that, it started increasing until minute 7.5, where it reached 100%, and was maintained for 2 min. At minute 11, initial conditions (100% of mobile phase A) were recovered and kept for 2 min for re-equilibration.

### Mass spectrometer

The SFC/LC chromatograph was coupled to a triple quadrupole mass spectrometer 8060 (Shimadzu Corporation, Kyoto, Japan). The study was carried out employing an ESI source. The interface temperature was set at 350 °C and 300 °C for LC and SFC, respectively. The desolvation line (DL) was set at 200 °C. Heat block was set at 400 °C. The interface voltage used was 4 kV. Regarding nebulizer, heating, and drying gas flows: 3 L/min, 10 L/min, and 10 L/min were used, respectively.

### Optimization of the MS/MS parameters

A Shimadzu extended multireaction monitoring (MRM) library was used for the development of the multiresidue method. As that feature shows many transitions for each pesticide; only three of them were selected following the sensitivity rank. Individual standard solutions of the pesticides were injected to confirm the transition with the highest signal (quantifier) and the second most sensitive (qualifier). Some compounds, such as internal standards (dimethoate-d6, carbendazim-d3, malathion-d10, and dichlorvos-d6), were not present in the library and had to be manually optimized using a precursor ion search. For proper identification, two transitions must be detected with an ion ratio difference less than 30% and a retention time drift below ± 0.1 min. Acquisition windows of ± 0.35 min were established for each pesticide. The 215 pesticides of the multiresidue method and their MS parameters appear on the Supplementary Information Table S1. The retention times observed in each chromatography are also described in Table S1.

## Results and discussion

### Ion source temperature evaluation

**Fig. 1:**
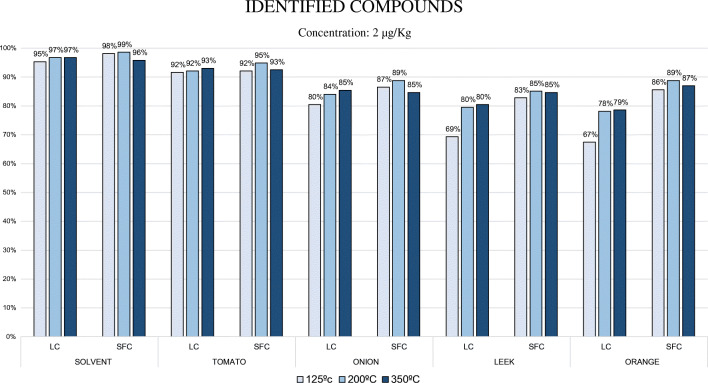
Percentage of identified compounds at the three ion source temperatures tested. The 215 pesticides were evaluated in solvent and tomato, onion, leek, and orange matrices by both techniques

The effect of the ESI interface temperatures was evaluated considering the sensitivity obtained keeping standard voltages (4 kV) and gas flows (nebulizer 3 L/min, heating gas 10 L/min, and drying gas 10 L/min). A majority of the compounds were correctly identified (two transitions with an adequate ion ratio) at the concentration level of 5 μg/kg by both techniques, and the study was focused at 2 μg/kg concentration level in spiked extracts of tomato, onion, leek, and orange to evaluate the differences in the number of compounds identified for each case. The identification criteria applied were those described in the SANTE document [27]. As can be observed in Figure 1, the interface temperatures tested were 350 °C, 200 °C, and 125 °C. Very similar results were obtained with pesticides in a pure solvent at the three temperatures studied: all the compounds were identified in the 95–99% range in both chromatographic methodologies. We had almost an identical situation with the tomato matrix, when 92–95% of the pesticides were identified at the different temperatures using both chromatographies. Regarding the onion matrix, there were no vast differences between techniques (80–89% range) being the results slightly better using SFC at 200 °C. In leek matrix, using LC, 80% of the compounds were identified at 200 °C and 350 °C; however, 69% of the compounds were identified at a lower temperature of 125 °C. In SFC, the percentages of identified compound in leek were between 83–85% in all the temperatures. A similar situation was found in the orange matrix analyzed by LC, with percentages of identified compounds at 200 °C and 350 °C of 78% and 79%, respectively. Analogous to what happened with the onion matrix, in orange, there was a decrease of identified compounds at 125 °C, where the percentage of identified compounds was 67%. Again, the interface temperatures using SFC did not have a strong impact on the results. The percentages of identified compounds in orange using this technic were in the 86–89% range in all the temperatures tested. Considering LC, the highest temperature provided better results in all the matrices, obtaining lower areas as the temperature decreased. This data confirms that high ESI interface temperature favors ionization and provides a general increase of sensitivity in the LC pesticide multiresidue methods. This increase of sensitivity of analytes at higher temperatures using LC coupled to ESI sources has been described many times before [28, 29]. Faster drying of the droplets allows reaching the Rayleigh limit faster, and therefore produces more progeny droplets at the same time. In the case of ESI droplets with high organic solvent content, as is the case of SFC and late eluting compounds in LC, a higher drying gas temperature does not increase this drying rate as much as it does for water-rich droplets [30]. Regarding SFC, although there were a higher number of identified compounds at 350 °C, a similar percentage was obtained at the lowest temperature tested (125 °C). Unlike LC, no significant sensitivity differences were observed between the three temperatures tested.

**Fig. 2:**
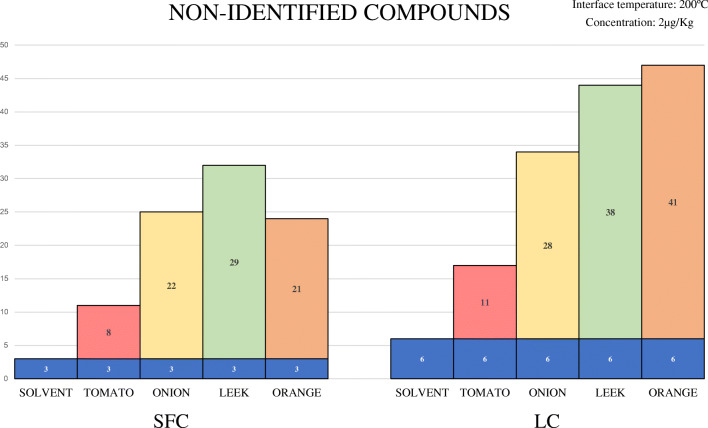
Number of non-identified compounds by SFC and LC in solvent and four different matrices at the concentration level of 2 μg/kg

Focusing on SFC area values, the differences between areas using the highest and lowest temperatures were lower than 30% in nearly all the compounds studied. Therefore, SFC did not show a very pronounced preference for higher or lower ESI interface temperature. This can bring additional benefits by analyzing some complex/thermolabile compounds at low temperatures, as previously demonstrated with captan and folpet [31]. Focusing on figure 2, we can observe the non-identified compounds per technique and matrix at the temperature test of 200 °C. Considering the number of pesticides not identified in each matrix, in LC we have an increase of approximately 30% per matrix compared to SFC, except in the orange matrix, where the non-identified compounds were almost twice in LC. Regarding solvent results, avermectin b1a was the only pesticide undetected in both chromatographies. The other two pesticides non-identified in SFC were cyazofamid and malathion (both presented isobaric interferences in the second transition). Considering LC, in addition to avermectin b1a, 5 pesticides were not identified: Bendiocarb and haloxyfop showed interferences in the second transition, iprodione presented low sensitivity and it decreased as the interface temperature was reduced; EPN and methamidophos also presented low sensitivity and they did not fulfill the ion ratio criteria at that low concentration level.

It is important to note that the spray emitter electrode used in the electrospray source is specifically designed for LC systems. Additional improvements in sensitivity can be expected by using a smaller diameter emitter electrode [32, 33]. Sampling efficiency in ESI using SFC is better due to the low flow reaching the source. In LC, the solvent flow is 300 μL/min; on the other hand, 75% of compounds in SFC elutes with a solvent flow below 140 μL/min. However, this mobile phase flow modification is not a dramatic reduction compared to LC typical flows. Moreover, there is a difference in the composition that arrives at the ESI source of the mobile phase depending on the type of chromatography. In the case of LC, the mobile phase starts at 100% water and increases to 100% of methanol during the elution, while in the case of SFC the elution in all cases is nearly 100% MeOH. Additionally, considering the gradient used in LC during the experiment, approximately 60% of the compounds eluted with 100% of methanol in the mobile phase and thus the inconveniences related to the presence of water during the ionization affected to a reduced percentage of pesticides in the multiresidue method. These factors could explain that even though the results were better using SFC, not huge sensitivity differences could be observed between both methodologies.

### Chemical groups and polarity

Another comparative study was performed in pure solvent to determine if there were physicochemical parameters that affected the sensitivity of compounds in each type of chromatography. A vial with a mix of pesticides at the concentration of 5 μg/L in solvent was injected in both techniques. The areas of the pesticides were compared, and those with a difference higher than 50% between methodologies were considered as more sensitive in the technic with the higher value and they were evaluated in terms of polarity and chemical group. Regarding the compounds in LC, 70 pesticides presented an area value higher than 50% compared to the same compound’s SFC values. From these compounds, there were a wide variety of chemical groups. However, organophosphates were the predominant group, with 19 pesticides (27%): azinphos-methyl, chlorpyirifos, demeton-s-methyl, diazinon, ethion, ethoprophos, fenthion, malathion, methidathion, phenthoate, phosmet, phoxim, pirimiphos-methyl, profenofos, prothiophos, and quinalphos. The following most prevalent groups were carbamates with 10 cases (carbaryl, fenobucarb, isoprocarb, methiocarb, methomyl, metolcarb, pirimicarb, promecarb, propoxur, XMC), urea derivatives herbicides with 7 pesticides (chlorbromuron, chlorotoluron, fenuron, isoproturon, linuron, metobromuron, and monolinuron), and triazines with 6 cases (atrazine, prometryn, propazine, simazine, terbuthylazine, terbutryn). The remaining pesticides had 3 or fewer matches per substance group. On the other hand, using SFC, 77 pesticides showed differences in their chromatographic areas higher than 50% compared to LC. In this case, triazoles were the most representative chemical group, with 17 compounds (22%): bromuconazole, cyproconazole, difenoconazole, diniconazole, epoxiconazole, fenbuconazole, fluquinconazole, flusilazole, flutriafol, hexaconazole, metconazole, myclobutanil, paclobutrazol, propiconazole, tebuconazole, tetraconazole, and triticonazole. Triazoles were followed by 11 organophosphates (demeton-s-methyl sulfone, EPN, fenamiphos, fenamiphos sulfone, fenamiphos sulfoxide, fenthion sulfone, fenthion sulfoxide, methamidophos, omethoate, and pyridaphention), 7 benzoylureas (chlorfluazuron, diflubenzuron, flufenoxuron, hexaflumuron, lufenuron, novaluron, and triflumuron), and 5 neonicotinoids (acetamiprid, imidacloprid, nitenpyram, thiacloprid, and thiamethoxam). The remaining chemical groups were not repeated more than 3 times.

It is already known that the compound elution in SFC does not follow the same trend as it does with LC [20]. While in reverse-phase LC the compounds elute in decreasing order of polarity, in SFC there are other factors involved in retention mechanisms like fluid density and modifier interactions [34], as supercritical CO_2_ is a non-polar mobile phase (a modifier gradient of MeOH gradually changes this situation). The pesticides that showed a higher sensitivity for each technique (70 pesticides in LC and 77 pesticides in the case of SFC) were evaluated using the octanol/water partition coefficient (*k*_ow_). The LogP_ow_ data were obtained from two different sources [35, 36]. The average LogP_ow_ of the most sensitive pesticides in SFC was 2.87. On the other hand, 3.27 was the average LogP_ow_ for those of LC. Considering a value of 3 as an average LogP_ow_ for those pesticides evaluated, the percentage of compounds below that value was 46% in LC and 44% in SFC. In the light of the results, logP_ow_ does not seem to be a parameter that influences sensitivity in one type of chromatography or another. Methomyl (0.09) and oxasulfuron (–0.81) were the pesticides with the lowest LogP_ow_ in LC and SFC, respectively. Bifenthrin was the pesticide with the highest LogP_ow_ in LC (6.60), while tau-fluvalinate was the pesticide with the highest LogP_ow_ in SFC (7.02).

### Ion suppression

Matrix effects were evaluated through two different experiments. First, matrix effects were calculated by comparing the slope values of the calibration curves in solvent with those values of the matrix-matched calibration curves [37]. Then, the total ion chromatograms (TIC) of each matrix were compared with the chromatograms of the corresponding multiresidue method. The aim of these experiments was to determine whether matrix ion suppression was also influenced by the different elution profiles in each chromatography. Matrix effects between 0 and 20% were considered low or non-existent; however, modifications of the signal between 20 and 50% and >50% were considered as medium and strong matrix effects, respectively. As exposed in Table 1, there were no big differences between types of chromatography in tomato matrix, with the results being slightly better in LC. However, focusing on the leek matrix, there was a huge difference in the percentage of compounds with a low or non-existent matrix effect, 5% and 28% for LC and SFC, respectively. Furthermore, the strong matrix effects in LC were 22% higher in comparison with SFC. As can be observed in the Table 1, the results in onion matrix were very similar to those in leek. Regarding the orange matrix, this difference was even more significant: 7% of compounds in LC had matrix effects below 20% while 53% of the pesticides showed low matrix effects in SFC. Our previous studies denoted that SFC analysis provides in general terms a low matrix effect due to its nebulization and sampling efficiency in the ion source [22, 38]. These data confirm that SFC is effective in reducing matrix effects in matrices with a high number of interfering components like onion, leek, and orange. However, in matrices like tomato, there were no vast differences.

**Table 1:** Percentage of the 215 pesticides affected by the matrix effects in tomato, leek, and orange matrices using reverse-phase liquid chromatography and supercritical fluid chromatography

	M.E.(%)	LC-MS/MS	SFC-MS/MS
Tomato	0–20	90%	83%
	20–50	8%	13%
	>50	1%	4%
	0–20	11%	29%
Onion (CELL)	20–50	28%	21%
	>50	61%	50%
Leek	0–20	5%	28%
	20–50	23%	22%
	>50	72%	50%
Orange	0–20	7%	53%
	20–50	53%	18%
	>50	39%	28%

**Fig. 3:**
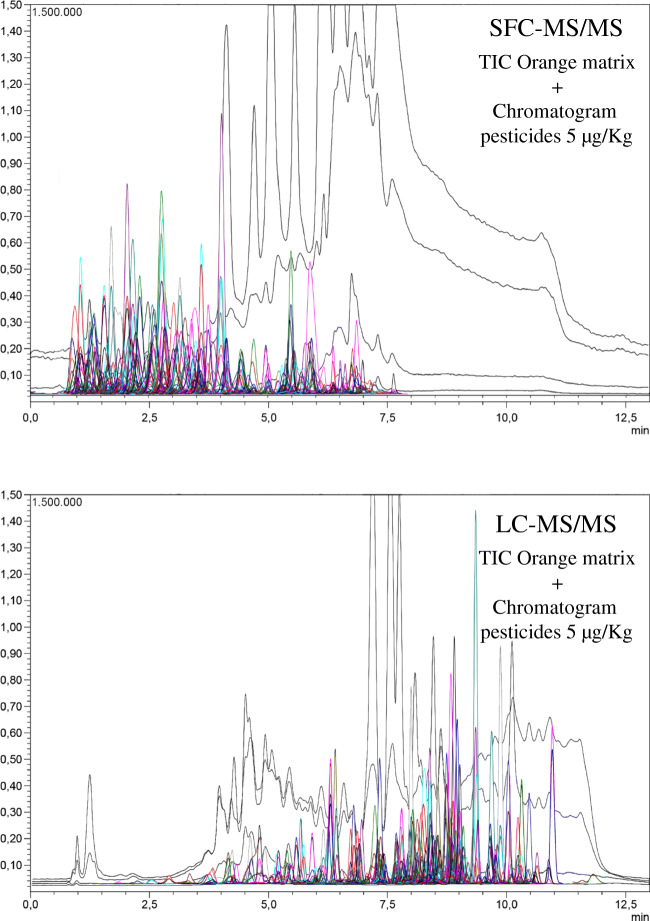
Total ion chromatograms (TIC) of orange matrix overlapped with the chromatograms of the multiresidue methods at the concentration level of 5 μg/kg

To evaluate the performance of both chromatographies against ion suppression, an injection of a blank extract of each matrix was carried out scanning the sample with the quadrupoles Q1 and Q3 and analyzing in the range of 100 to 1000 m/z (positive and negative polarity). The total ion chromatograms (TIC) were then overlapped with the chromatograms of the multiresidue methods. The analytes overlapping with the TIC of tomato, onion, and leek were challenging to evaluate. Based on the intensity of the TICs in matrix, the interactions between the analytes and the interferents of tomato, onion, and leek were similar in both methods. Tomato TIC intensity was low compared to that of the other two matrices, and no strong interference of the TIC with the compounds was observed. The opposite situation was found in leek and onion matrices, where a huge TIC intensity overlapped most of the pesticide’s signals in both techniques. However, the most interesting case involved the orange matrix, where the matrix effect differences were higher. In Figure 3, both TIC and multiresidue chromatograms have been equally scaled. At first sight, one may think that matrix effects should be higher in SFC due to the high TIC intensity. However, 75% of SFC compounds eluted before minute 5 (the last compound eluted at 7.622 min), and the intensity of the TIC in that region is approximately half compared to the compound elution zone in the LC chromatogram. This approach implies that the reduction of matrix effects in SFC is not only related to its sampling efficiency in the ESI source. The different elution mechanisms between technics play an important role in matrix effects as well. The data of identified compounds (see subsection “Ion source temperature evaluation”) and matrix effects in leek and onion showed better results in SFC compared to LC. Furthermore, in orange, this enhancement was even stronger. For example, azinphos-ethyl, diniconazole, fenpropathrin, and propiconazole were not identified at 350 °C in leek and orange matrices using LC. Regarding the SFC results for these pesticides, they were not identified in leek, but they were identified in orange matrix. All these compounds have retention times ranging between minutes 1 and 4, which means that they eluted with the lowest amount of coextracted matrix compounds in SFC. This evidence suggests that the good results of the orange matrix were thanks to a combination of the ionization efficiency and reduction of ion suppression due to the decrease of coelution with matrix components.

## Conclusions

A comparison of two types of chromatography has been made using the same ESI-MS platform. Different ion source temperatures were studied: LC needed the highest temperature tested (350 °C) to achieve the highest sensitivity. However, in SFC, the sensitivity differences were much lower between the higher and the lower temperatures tested. In terms of sensitivity, SFC showed better results in complex matrices like leek, onion, and orange. Focusing on the compounds that presented higher sensitivity in all the studied matrices in each technic, it was observed that triazoles were the chemical group whose sensitivity was higher in SFC, followed by organophosphates. In LC, a majority correspond to organophosphates. The most sensitive pesticides in each technic were studied in terms of their octanol/water partition coefficient and the values were similar in both cases, concluding that this parameter is not decisive in the prediction of which chromatography is the best for each pesticide. Matrix effects were evaluated in terms of ion suppression. Higher ion suppression was observed in LC when complex matrices were analyzed. Furthermore, a test comparing the TIC of the matrices proved that the different elution mechanisms play an important factor in the reduction of ion suppression. Considering the results, SFC provides a better performance regarding LC when the coextracts from the matrix are very high. Furthermore, the SFC advantages are also of interest when low ion source temperatures are necessary for thermolabile compound analysis.

## Supplementary Information

ESM 1 (DOCX 48 kb)
