# Supplementary material for: Liquid chromatography versus supercritical fluid chromatography coupled to mass spectrometry: a comparative study of performance for multiresidue analysis of pesticides
Source: Anal Bioanal Chem. 2021 Jul 29;413(23):5849–57. doi: 10.1007/s00216-021-03565-4 (PMC8437864; doi:10.1007/s00216-021-03565-4)
Supplement: Supplementary file 1 — (DOCX 48 kb) [file 216_2021_3565_MOESM1_ESM.docx]

**Table S1** Compounds analyzed and their corresponding acquisition parameters.

| **Compound** | **Target (1)** | **Target (2)** | **Target CE** | **Ref.(1)** | **Ref.(2)** | **Ref. CE** | **RT SFC** | **RT LC** |
| --- | --- | --- | --- | --- | --- | --- | --- | --- |
| Acetamiprid | 223.1 | 126.1 | -21 | 223.1 | 99.1 | -39 | 6.4 | 5.1 |
| Alachlor | 270.1 | 162.2 | -21 | 270.1 | 147.1 | -31 | 1.3 | 7.7 |
| Ametoctradin | 276.1 | 176 | -38 | 276.1 | 149.2 | -37 | 6.7 | 8.4 |
| Anilofos | 368 | 125 | -32 | 368 | 199.1 | -15 | 2.4 | 8.2 |
| Atrazine | 216.1 | 174.1 | -18 | 216.1 | 104.1 | -30 | 3.6 | 7 |
| Avermectin B1a | 890.5 | 305.2 | -29 | 890.5 | 567.2 | -15 | 6.5 | 9.1 |
| Azinphos-ethyl | 346 | 97 | -33 | 346 | 132.1 | -17 | 3.1 | 8.2 |
| Azinphos-methyl | 318 | 77 | -36 | 318 | 132.1 | -16 | 3.5 | 7.8 |
| Azoxystrobin | 404 | 372 | -15 | 404 | 329 | -31 | 4.2 | 7.1 |
| Benalaxyl | 326.2 | 148.1 | -21 | 326.2 | 91.1 | -37 | 1.8 | 7.9 |
| Bendiocarb | 224.1 | 109 | -19 | 224.1 | 81.1 | -34 | 2 | 4.3 |
| Bifenazate | 301.1 | 198.1 | -11 | 301.1 | 170 | -19 | 4.7 | 8 |
| Bifenazate_diazene | 299 | 213.1 | -13 | 299 | 197 | -13 | 2.8 | 9 |
| Bifenthrin | 440.2 | 181.2 | -17 | 440.2 | 165.1 | -55 | 1.7 | 10.5 |
| Boscalid | 343 | 307 | -21 | 343 | 272 | -32 | 6.1 | 7.7 |
| Bromacil | 261 | 204.9 | -14 | 261 | 188 | -28 | 5.2 | 6.3 |
| Bromuconazole | 375.9 | 159 | -31 | 379.9 | 161.1 | -29 | 4.3 | 7.8 |
| Bupirimate | 317.2 | 166 | -24 | 317.2 | 108 | -26 | 1.9 | 7.8 |
| Buprofezin | 306.2 | 201.1 | -12 | 306.2 | 116.2 | -16 | 2.2 | 8.9 |
| Carbaryl | 202.1 | 145.1 | -12 | 202.1 | 127 | -25 | 5.1 | 6.8 |
| Carbendazim | 192.1 | 160.2 | -19 | 192.1 | 132.2 | -30 | 5.5 | 4.7 |
| Chlorantraniliprole | 483.9 | 452.9 | -19 | 483.9 | 285.9 | -17 | 6.8 | 7.1 |
| Chlorbromuron | 295 | 205.9 | -19 | 295 | 182.1 | -17 | 4.5 | 8.1 |
| Chlorfenvinphos | 359 | 170 | -39 | 359 | 155.2 | -13 | 1.5 | 7.9 |
| Chlorfluazuron | 539.9 | 382.9 | -20 | 539.9 | 158 | -21 | 6.2 | 10.1 |
| Chloridazon | 222.1 | 104.1 | -22 | 222.1 | 92.2 | -26 | 7.1 | 5.5 |
| Chlorotoluron | 213.1 | 72.2 | -22 | 213.1 | 140 | -24 | 6 | 6.8 |
| Chloroxuron | 291.1 | 72.2 | -23 | 291.1 | 218.1 | -26 | 6.6 | 7.9 |
| Chlorpyrifos | 350 | 198 | -21 | 352 | 199.9 | -20 | 1.5 | 9.6 |
| Chromafenozide | 394.9 | 175.2 | -21 | 394.9 | 91.1 | -55 | 6.3 | 7.4 |
| Clofentezine | 303 | 138.2 | -15 | 303 | 102.1 | -37 | 4.4 | 9.6 |
| Clomazone | 239.9 | 125 | -21 | 241.9 | 127 | -21 | 1.4 | 7.1 |
| Coumaphos | 363 | 227 | -26 | 363 | 306.8 | -19 | 3.4 | 8.7 |
| Cyazofamid | 325 | 108.1 | -15 | 325 | 261 | -10 | 3 | 8.3 |
| Cyflufenamid | 413.1 | 295.1 | -16 | 413.1 | 241 | -24 | 1.3 | 8.4 |
| Cyhalofop-butyl | 375.2 | 256 | -16 | 375.2 | 120 | -32 | 1.6 | 8.8 |
| Cymoxanil | 199.1 | 128.2 | -9 | 199.1 | 111.2 | -19 | 1.2 | 5.4 |
| Cypermethrin | 433.1 | 191 | -16 | 435.1 | 193.1 | -16 | 2.4 | 10 |
| Cyproconazole | 292.1 | 125.1 | -32 | 292.1 | 89 | -54 | 4.7 | 7.6 |
| Cyprodinil | 226.1 | 93 | -37 | 226.1 | 108 | -27 | 4.3 | 8.9 |
| Deet | 192.1 | 119 | -17 | 192.1 | 91 | -30 | 1.4 | 6.3 |
| Deltamethrin | 523 | 281 | -17 | 523 | 505.9 | -11 | 3.7 | 10.1 |
| Demeton-S-methyl | 231.1 | 89 | -9 | 231.1 | 61.1 | -30 | 1.2 | 5.9 |
| Demeton-S-methyl-sulfone | 263 | 169.1 | -16 | 263 | 109.1 | -28 | 1.5 | 4.2 |
| Desethyl_terbuthylazine | 202 | 146.1 | -18 | 202 | 104 | -27 | 4.9 | 8 |
| Diazinon | 305.1 | 169.1 | -22 | 305.1 | 97 | -36 | 1.1 | 5.9 |
| Dichlorvos | 238 | 220.9 | -11 | 238 | 109.1 | -21 | 0.8 | 4.3 |
| Dicrotophos | 237.9 | 112.2 | -13 | 237.9 | 127 | -16 | 1.3 | 7.1 |
| Diethofencarb | 268.2 | 124.2 | -31 | 268.2 | 226.1 | -10 | 2.2 | 8.6 |
| Difenoconazole | 406.1 | 250.9 | -25 | 406.1 | 188 | -45 | 4.3 | 7 |
| Difenoxuron | 287.1 | 72.1 | -22 | 287.1 | 123.2 | -21 | 6.5 | 8.7 |
| Diflubenzuron | 311 | 158.1 | -16 | 311 | 141.1 | -32 | 5.3 | 5.2 |
| Dimethoate | 230 | 198.9 | -10 | 230 | 125 | -21 | 3.6 | 7.3 |
| Dimethomorph | 388.1 | 301 | -21 | 388.1 | 165.1 | -34 | 4.4 | 8.5 |
| Diniconazole | 326.1 | 70.1 | -25 | 328 | 70 | -25 | 4.1 | 6.6 |
| Diuron | 233 | 72.1 | -23 | 233 | 46.2 | -17 | 6.6 | 7.6 |
| Dodine | 228.3 | 57.1 | -25 | 228.3 | 71.1 | -23 | 7 | 6.8 |
| Edifenphos | 311 | 109 | -32 | 311 | 111.1 | -22 | 3.4 | 8.1 |
| Emamectin B1a | 886.4 | 158.2 | -36 | 886.4 | 82.1 | -55 | 6.7 | 8 |
| EPN | 324.1 | 295.9 | -14 | 324.1 | 157 | -30 | 2.8 | 9.4 |
| Epoxiconazole | 330 | 121.1 | -21 | 330 | 101.1 | -44 | 2.7 | 8 |
| Ethion | 385 | 143 | -25 | 385 | 198.9 | -10 | 1.5 | 9.3 |
| Ethiprole | 397 | 350.9 | -22 | 397 | 254.9 | -36 | 4.5 | 7.2 |
| Ethirimol | 210.2 | 98.2 | -27 | 210.2 | 140.2 | -21 | 5.6 | 5.2 |
| Ethoprophos | 243.1 | 131 | -21 | 243.1 | 172.9 | -15 | 1.2 | 7.3 |
| Etofenprox | 394.2 | 177.1 | -14 | 394.2 | 107.1 | -41 | 3.8 | 10.8 |
| Etoxazole | 360.1 | 141.1 | -30 | 360.1 | 113.1 | -55 | 2.1 | 9.2 |
| Famoxadone | 392.2 | 331.1 | -10 | 392.2 | 238 | -18 | 6.3 | 8.9 |
| Fenamidone | 312.1 | 92.1 | -26 | 312.1 | 236 | -15 | 5.5 | 7.6 |
| Fenamiphos | 304.1 | 217 | -23 | 304.1 | 202 | -36 | 3 | 7.6 |
| Fenamiphos-sulfone | 336.1 | 266 | -20 | 336.1 | 188 | -28 | 3.5 | 5.8 |
| Fenamiphos-sulfoxide | 320.1 | 233 | -26 | 320.1 | 108 | -41 | 4.4 | 5.4 |
| Fenarimol | 331 | 268 | -23 | 331 | 189 | -48 | 5.6 | 8.1 |
| Fenazaquin | 307.2 | 161.1 | -17 | 307.2 | 131.2 | -45 | 3.8 | 10.1 |
| Fenbuconazole | 337.1 | 70.1 | -22 | 337.1 | 125.1 | -29 | 4.7 | 8 |
| Fenhexamid | 302.1 | 97.1 | -24 | 302.1 | 143 | -34 | 6.8 | 8 |
| Fenobucarb | 208.1 | 95 | -15 | 208.1 | 152.1 | -9 | 2.1 | 7.1 |
| Fenoxycarb | 302.1 | 116.2 | -11 | 302.1 | 256.1 | -13 | 3.2 | 8.3 |
| Fenpropathrin | 367 | 125.1 | -17 | 367 | 350.1 | -8 | 1.7 | 9.7 |
| Fenpropimorph | 304.2 | 117 | -55 | 304.2 | 147.1 | -29 | 1.4 | 6.3 |
| Fenpyroximate | 422.1 | 366 | -17 | 422.1 | 138.1 | -32 | 2.9 | 9.6 |
| Fenthion | 278.9 | 169 | -17 | 278.9 | 105.2 | -26 | 1.8 | 8.9 |
| Fenthion-sulfone | 311 | 125.1 | -23 | 311 | 109 | -27 | 2 | 6.7 |
| Fenthion-sulfoxide | 295 | 279.9 | -19 | 295 | 109 | -32 | 2.9 | 6.1 |
| Fenuron | 165 | 72.2 | -22 | 165 | 77 | -32 | 5 | 5.1 |
| Fipronil | 435 | 330 | 16 | 435 | 250.1 | 27 | 3.5 | 8 |
| Flazasulfuron | 408 | 182.1 | -21 | 408 | 139 | -40 | 5.8 | 7.1 |
| Fluacrypyrim | 427.2 | 145.2 | -24 | 427.2 | 205.1 | -11 | 1.2 | 8.3 |
| Fluazifop | 327.7 | 282 | -18 | 327.7 | 254 | -28 | 5.2 | 7.2 |
| Flucythrinate | 469.2 | 412 | -13 | 469.2 | 181 | -36 | 2 | 9.5 |
| Fludioxonil | 247 | 180.2 | 28 | 247 | 169.2 | 31 | 6.7 | 8.3 |
| Flufenacet | 364.1 | 152.1 | -19 | 364.1 | 194 | -12 | 1 | 7.5 |
| Flufenoxuron | 489 | 140.9 | -46 | 489 | 158.1 | -21 | 5.3 | 9.8 |
| Fluopicolide | 383 | 172.9 | -23 | 383 | 145 | -50 | 3.2 | 7.6 |
| Fluopyram | 397 | 208 | -23 | 397 | 145 | -54 | 1.7 | 7.5 |
| Fluquinconazole | 376 | 307 | -29 | 376 | 349 | -20 | 3.7 | 8.3 |
| Flusilazole | 316.1 | 247 | -18 | 316.1 | 165.1 | -26 | 2.3 | 8 |
| Flutriafol | 302.1 | 70.1 | -22 | 302.1 | 123 | -26 | 3.6 | 6.7 |
| Fluxapyroxad | 382 | 342 | -22 | 382 | 313.9 | -24 | 2.7 | 7.6 |
| Fosthiazate | 284.1 | 104.1 | -21 | 284.1 | 227.9 | -11 | 1.7 | 6.2 |
| Haloxyfop | 362.1 | 316 | -19 | 364.1 | 318.2 | -18 | 5.3 | 8 |
| Hexaconazole | 314.1 | 70 | -22 | 314.1 | 159 | -30 | 3.1 | 8.2 |
| Hexaflumuron | 458.8 | 439 | 11 | 458.8 | 175.1 | 37 | 5.3 | 9.3 |
| Hexythiazox | 353.1 | 228 | -16 | 353.1 | 168 | -24 | 2.8 | 9.5 |
| Imazalil | 297.1 | 159.1 | -23 | 297.1 | 200.9 | -19 | 4.1 | 5.9 |
| Imidacloprid | 256.1 | 175 | -20 | 256.1 | 209 | -17 | 6.9 | 5 |
| Indoxacarb | 527.7 | 203 | -37 | 527.7 | 218 | -24 | 1.7 | 8.6 |
| Ioxynil | 369.8 | 127.1 | 36 | 369.8 | 215 | 33 | 7.5 | 8.3 |
| Iprodione | 330.1 | 245 | -15 | 332.1 | 247 | -15 | 2.2 | 8.4 |
| Iprovalicarb | 321.2 | 119.2 | -21 | 321.2 | 203 | -9 | 1.9 | 7.2 |
| Isoprocarb | 194.1 | 95 | -16 | 194.1 | 137 | -13 | 2.1 | 6.6 |
| Isoprothiolane | 290.8 | 188.9 | -22 | 290.8 | 231 | -12 | 2.1 | 7.5 |
| Isoproturon | 207.2 | 72.2 | -23 | 207.2 | 165 | -14 | 4.5 | 6.7 |
| Isoxaflutole | 360.1 | 251 | -16 | 360.1 | 144 | -54 | 1 | 7.1 |
| Kresoxim-methyl | 314.1 | 267 | -8 | 314.1 | 235 | -16 | 1.7 | 8.3 |
| Lenacil | 234.9 | 153.2 | -16 | 234.9 | 136 | -30 | 5.9 | 6.7 |
| Linuron | 248.8 | 160 | -18 | 248.8 | 182.1 | -16 | 3.8 | 7.9 |
| Lufenuron | 508.9 | 326 | 19 | 508.9 | 339 | 12 | 4.5 | 9.5 |
| Malathion | 348.1 | 127.1 | -17 | 348.1 | 330.9 | -8 | 1 | 7.5 |
| Mandipropamid | 412.1 | 327.9 | -15 | 412.1 | 125.1 | -36 | 5 | 7.5 |
| Mepanipyrim | 224.1 | 106.1 | -26 | 224.1 | 104.1 | -28 | 4.4 | 8.6 |
| Metaflumizone | 506.8 | 178.1 | -27 | 506.8 | 287 | -25 | 5.3 | 9.1 |
| Metalaxyl | 280.1 | 220 | -14 | 280.1 | 192.1 | -18 | 1.2 | 6.2 |
| Metconazole | 320.1 | 70.2 | -23 | 320.1 | 125.1 | -35 | 5.3 | 8.4 |
| Methamidophos | 142.2 | 94 | -15 | 142.2 | 124.9 | -16 | 4.9 | 1.8 |
| Methidathion | 320 | 145 | -12 | 320 | 302.8 | -7 | 1.8 | 7.5 |
| Methiocarb | 226.1 | 169.1 | -10 | 226.1 | 121.1 | -19 | 3.7 | 7.6 |
| Methiocarb-sulfone | 275.1 | 122.1 | -23 | 275.1 | 258 | -9 | 5 | 5.3 |
| Methiocarb-sulfoxide | 242.1 | 185.1 | -14 | 242.1 | 122.1 | -28 | 5.6 | 4.8 |
| Methomyl | 163 | 87.9 | -10 | 163 | 106.2 | -11 | 4.1 | 4.3 |
| Methoxyfenozide | 369.2 | 149.2 | -18 | 369.2 | 91.2 | -47 | 6.2 | 7.2 |
| Metobromuron | 259 | 170 | -19 | 259 | 148.1 | -15 | 2.8 | 7.2 |
| Metolachlor | 284.1 | 252.1 | -16 | 284.1 | 176.1 | -26 | 1.4 | 7.6 |
| Metolcarb | 166.1 | 109.1 | -12 | 166.1 | 94.1 | -30 | 2.2 | 6 |
| Metrafenone | 409 | 209.1 | -16 | 409 | 227 | -23 | 3.5 | 8.7 |
| Monocrotophos | 240.9 | 127.1 | -21 | 240.9 | 193 | -12 | 2.8 | 4.2 |
| Monolinuron | 215.1 | 99.1 | -34 | 215.1 | 148 | -15 | 2.3 | 6.9 |
| Monuron | 199.1 | 72.2 | -22 | 199.1 | 126 | -26 | 6 | 6.3 |
| Myclobutanil | 289.1 | 70.1 | -22 | 289.1 | 125 | -31 | 2.6 | 7.4 |
| Neburon | 274.8 | 88 | -17 | 274.8 | 114.2 | -15 | 6.3 | 8.3 |
| Nitenpyram | 271.1 | 225 | -12 | 271.1 | 237 | -19 | 6.5 | 4 |
| Novaluron | 493 | 158 | -18 | 493 | 141.1 | -40 | 3.7 | 8.9 |
| Omethoate | 214.1 | 125 | -23 | 214.1 | 183 | -11 | 2.4 | 3 |
| Oxadiargyl | 358.1 | 340.9 | -11 | 358.1 | 223 | -21 | 1.6 | 8.7 |
| Oxadixyl | 296.2 | 219.1 | -15 | 296.2 | 279.1 | -8 | 3 | 5.4 |
| Oxamyl | 237.1 | 72.1 | -22 | 237.1 | 90 | -8 | 4.6 | 4 |
| Oxasulfuron | 407.1 | 150.2 | -21 | 407.1 | 107 | -46 | 6.2 | 6 |
| Paclobutrazol | 294.1 | 70.1 | -22 | 294.1 | 125.1 | -36 | 3.2 | 7.3 |
| Paraoxon-methyl | 248 | 202 | -20 | 248 | 109 | -29 | 1.4 | 5.8 |
| Penconazole | 284.1 | 159 | -30 | 284.1 | 123 | -50 | 2.4 | 8.1 |
| Pencycuron | 329.1 | 125 | -25 | 329.1 | 89 | -55 | 6.1 | 8.8 |
| Pendimethalin | 282.2 | 212 | -11 | 282.2 | 194 | -18 | 1.3 | 9.8 |
| Permethrin | 408.1 | 183.1 | -21 | 410.1 | 183 | -21 | 2.6 | 10.8 |
| Phenothrin | 351.2 | 183.1 | -21 | 351.2 | 128 | -54 | 2 | 10.3 |
| Phenthoate | 321 | 79.1 | -41 | 321 | 246.9 | -12 | 1.4 | 8.3 |
| Phosalone | 368 | 182 | -17 | 368 | 111 | -38 | 2.6 | 8.9 |
| Phosmet | 335 | 160.1 | -16 | 335 | 317.9 | -8 | 2.8 | 7.8 |
| Phoxim | 299 | 77.1 | -30 | 299 | 129.1 | -12 | 1.3 | 8.7 |
| Pirimicarb | 239.2 | 182.1 | -16 | 239.2 | 85.1 | -28 | 1.5 | 5.3 |
| Pirimicarb-desmethyl | 225.1 | 168.1 | -14 | 225.1 | 180 | -14 | 2.4 | 4.4 |
| Pirimiphos-methyl | 306.1 | 164.1 | -23 | 306.1 | 108.2 | -31 | 1.4 | 8.6 |
| Prochloraz | 376 | 308 | -13 | 376 | 70 | -26 | 4.1 | 8.3 |
| Profenofos | 375 | 304.7 | -19 | 375 | 346.8 | -13 | 1.8 | 8.8 |
| Promecarb | 208.1 | 109.1 | -15 | 208.1 | 151.2 | -10 | 2.1 | 7.4 |
| Prometryn | 242.1 | 158 | -22 | 242.1 | 200.2 | -19 | 3 | 7.6 |
| Propaquizafop | 443.8 | 100.2 | -22 | 443.8 | 299 | -24 | 3.6 | 9.2 |
| Propargite | 368.2 | 175.1 | -16 | 368.2 | 231.1 | -11 | 1.8 | 9.5 |
| Propazine | 230.2 | 146.2 | -23 | 230.2 | 188.1 | -18 | 2.6 | 7.5 |
| Propiconazole | 342 | 158.9 | -28 | 342 | 69.1 | -22 | 2.3 | 8.1 |
| Propoxur | 209.9 | 111.2 | -14 | 209.9 | 93.1 | -24 | 2 | 5.9 |
| Propyzamide | 256 | 190 | -14 | 256 | 173 | -22 | 3.1 | 7.9 |
| Proquinazid | 373.1 | 330.9 | -16 | 373.1 | 288.8 | -25 | 3.2 | 10.1 |
| Prosulfocarb | 252.1 | 91 | -23 | 252.1 | 128.1 | -12 | 1.5 | 8.8 |
| Prothiophos | 345 | 240.9 | -20 | 347 | 242.8 | -16 | 2.1 | 10.4 |
| Pymetrozine | 218.1 | 105 | -22 | 218.1 | 78.1 | -42 | 6.7 | 3.5 |
| Pyraclostrobin | 388 | 194.1 | -13 | 388 | 133 | -35 | 4.4 | 8.7 |
| Pyridaben | 365.2 | 147.1 | -25 | 365.2 | 308.9 | -13 | 4.2 | 9.9 |
| Pyridalyl | 491.9 | 111.1 | -29 | 491.9 | 109.1 | -30 | 2.8 | 11.7 |
| Pyridaphenthion | 341.1 | 189 | -22 | 341.1 | 204.9 | -22 | 2.3 | 7.5 |
| Pyridate | 379.1 | 207 | -18 | 379.1 | 104.2 | -39 | 4 | 10.3 |
| Pyrimethanil | 200.1 | 107 | -24 | 200.1 | 183 | -24 | 3.5 | 7.8 |
| Pyriproxyfen | 322.1 | 96.1 | -16 | 322.1 | 185 | -24 | 3.4 | 9.7 |
| Quinalphos | 299.1 | 163 | -22 | 299.1 | 147.1 | -22 | 1.6 | 8.5 |
| Quinoclamine | 208 | 105.1 | -24 | 208 | 89 | -41 | 6.2 | 7.1 |
| Quinoxyfen | 308 | 197 | -31 | 308 | 272 | -28 | 3.8 | 10 |
| Quizalofop (free acid) | 345 | 299 | -19 | 345 | 244 | -30 | 6.8 | 8.2 |
| Quizalofop-ethyl | 373.1 | 298.9 | -19 | 373.1 | 270.9 | -26 | 2.9 | 9.2 |
| Rotenone | 395.1 | 213 | -23 | 395.1 | 192 | -24 | 5.2 | 8.4 |
| Simazine | 202 | 104.1 | -26 | 202 | 96.1 | -26 | 4.3 | 6.8 |
| Spirodiclofen | 411.1 | 313.1 | -14 | 411.1 | 294.9 | -25 | 2.1 | 9.5 |
| Spiromesifen | 371.2 | 273.1 | -15 | 371.2 | 255.2 | -23 | 1.7 | 9 |
| Spirotetramat | 374.1 | 216 | -33 | 374.1 | 302 | -18 | 4.4 | 7.1 |
| Tau-Fluvalinate | 503.1 | 181 | -29 | 503.1 | 208.1 | -13 | 2.1 | 10.2 |
| Tebuconazole | 308.2 | 70.1 | -22 | 308.2 | 125.1 | -38 | 4.8 | 8.1 |
| Tebufenozide | 353.2 | 133.1 | -21 | 353.2 | 297 | -9 | 5.8 | 7.6 |
| Tebufenpyrad | 334.2 | 117 | -35 | 334.2 | 145.2 | -27 | 2.4 | 8.8 |
| Teflubenzuron | 378.8 | 339 | 12 | 378.8 | 358.9 | 7 | 5.7 | 9.7 |
| Terbuthylazine | 230.1 | 174.1 | -17 | 230.1 | 104 | -33 | 3 | 7.6 |
| Terbuthylazine-desethyl | 202.1 | 146.1 | -16 | 202.1 | 104.1 | -28 | 4.9 | 6.8 |
| Terbutryn | 242.1 | 158 | -25 | 242.1 | 90.9 | -27 | 3 | 7.6 |
| Tetraconazole | 372 | 159 | -30 | 372 | 70.1 | -22 | 1.7 | 7.8 |
| Tetramethrin | 332.2 | 164.1 | -24 | 332.2 | 135.1 | -18 | 1.4 | 8.9 |
| Thiabendazole | 201.8 | 175 | -25 | 201.8 | 131.2 | -32 | 7.2 | 5.4 |
| Thiacloprid | 252.8 | 126.1 | -21 | 252.8 | 90.1 | -39 | 7 | 5.6 |
| Thiamethoxam | 292 | 211.1 | -13 | 292 | 132 | -22 | 6.6 | 4.5 |
| Thiobencarb | 257.8 | 125.1 | -21 | 257.8 | 89 | -50 | 1.7 | 8.7 |
| Triazophos | 314.1 | 162.1 | -19 | 314.1 | 119.2 | -34 | 2.9 | 7.8 |
| Tricyclazole | 190.1 | 136 | -29 | 190.1 | 109 | -36 | 6.3 | 5.6 |
| Trifloxystrobin | 408.8 | 186.1 | -19 | 408.8 | 145.1 | -44 | 1.3 | 8.6 |
| Triflumizole | 346 | 278 | -11 | 348 | 280 | -11 | 2 | 8.6 |
| Triflumuron | 359 | 156.1 | -17 | 359 | 139.1 | -30 | 3.9 | 8.8 |
| Triticonazole | 318.1 | 70.2 | -22 | 318.1 | 125 | -28 | 5.5 | 7.6 |
| Tritosulfuron | 446 | 194.9 | -21 | 446 | 145.1 | -35 | 4.8 | 7.2 |
| XMC (3,5-xylyl methylcarbamate) | 180.1 | 123.1 | -12 | 180.1 | 108 | -28 | 2.2 | 6.6 |
| Zoxamide | 336 | 187 | -23 | 336 | 159 | -40 | 5.8 | 8.7 |
